# Aldosterone Decreases Vasopressin-Stimulated Water Reabsorption in Rat Inner Medullary Collecting Ducts

**DOI:** 10.3390/cells9040967

**Published:** 2020-04-14

**Authors:** Yanhua Wang, Fuying Ma, Eva L. Rodriguez, Janet D. Klein, Jeff M. Sands

**Affiliations:** Renal Division, Department of Medicine, Emory University School of Medicine, Atlanta, GA 30322, USA; ywang68@emory.edu (Y.W.); fuying.ma@emory.edu (F.M.); eva.rodriguez@emory.edu (E.L.R.); janet.klein@emory.edu (J.D.K.)

**Keywords:** mineralocorticoid receptor, natriuresis, non-genomic, AQP2, urea transport

## Abstract

Aldosterone indirectly regulates water reabsorption in the distal tubule by regulating sodium reabsorption. However, the direct effect of aldosterone on vasopressin-regulated water and urea permeability in the rat inner medullary collecting duct (IMCD) has not been tested. We investigated whether aldosterone regulates osmotic water permeability in isolated perfused rat IMCDs. Adding aldosterone (500 nM) to the bath significantly decreased osmotic water permeability in the presence of vasopressin (50 pM) in both male and female rat IMCDs. Aldosterone significantly decreased aquaporin-2 (AQP2) phosphorylation at S256 but did not change it at S261. Previous studies show that aldosterone can act both genomically and non-genomically. We tested the mechanism by which aldosterone attenuates osmotic water permeability. Blockade of gene transcription with actinomycin D did not reverse aldosterone-attenuated osmotic water permeability. In addition to AQP2, the urea transporter UT-A1 contributes to vasopressin-regulated urine concentrating ability. We tested aldosterone-regulated urea permeability in vasopressin-treated IMCDs. Blockade of gene transcription did not reverse aldosterone-attenuated urea permeability. In conclusion, aldosterone directly regulates water reabsorption through a non-genomic mechanism. Aldosterone-attenuated water reabsorption may be related to decreased trafficking of AQP2 to the plasma membrane. There may be a sex difference apparent in the inhibitory effect of aldosterone on water reabsorption in the inner medullary collecting duct. This study is the first to show a direct effect of aldosterone to inhibit vasopressin-stimulated osmotic water permeability and urea permeability in perfused rat IMCDs.

## 1. Introduction

Aldosterone is a steroid hormone that is produced in the adrenal cortex. Its major renal effect is to regulate electrolyte and water homeostasis in the distal tubule, thus maintaining blood pressure and extracellular fluid homeostasis through the activation of mineralocorticoid receptors (MR) in epithelial cells [1]. Aldosterone enters an epithelial cell and binds to the MR. The complex of aldosterone and MR translocates into the nucleus and regulates gene transcription of, among others, the epithelial sodium channel (ENaC) and the signaling proteins and kinases that impact channel and transporter activity, such as serum/glucocorticoid kinases (SGKs). This results in increased apical membrane accumulation and activity of ENaC, thus increasing sodium reabsorption and subsequent water reabsorption (reviewed in [2,3]).

In addition to the genomic effects, aldosterone has rapid actions that are independent of transcription and translation. Aldosterone can have rapid effects on sodium chloride cotransport (NCC) in distal convoluted tubules [4], bicarbonate reabsorption in proximal tubules [5], natriuresis [6], and several signaling processes in inner medullary collecting ducts (IMCDs) and other nephron segments [7,8,9,10]. Aldosterone increases calcineurin activity in rat cortical collecting ducts through an MR-dependent but transcription-independent mechanism [11]. Our published data show that inhibiting calcineurin alters the phosphorylation and the activity of the urea transporter UT-A1 in the IMCD [12,13].

Aldosterone has a complex interaction with vasopressin (AVP). Vasopressin is the major hormone regulating water permeability in the kidney [14]. Vasopressin binds to V2 receptors on the IMCD basolateral plasma membrane, resulting in stimulation of adenylyl cyclase and cyclic AMP (cAMP) generation. Stimulation of the cAMP dependent protein kinase A (PKA) promotes phosphorylation of key transporters and channels, resulting in an increase in water permeability [15]. Vasopressin stimulation of water permeability is due, in large part, to stimulation of the aquaporin 2 (AQP2) water channel [16,17]. AQP2, located at the apical plasma membrane and the subapical vesicles in collecting duct principal cells, is regulated by vasopressin-stimulated increases in both its phosphorylation and its apical plasma membrane accumulation [18]. Through the cAMP-PKA pathway, vasopressin also increases urea transport by stimulating UT-A1 [14,19,20].

Although aldosterone indirectly regulates water reabsorption in distal tubules, its effect on vasopressin-regulated water and urea permeability in rat IMCDs has not been tested, nor has whether there is a sex difference been examined. Our published data show that aldosterone decreases UT-A1 protein in the inner medullary tip [21]. Nielsen et al. reported that aldosterone increases urine production and decreases apical membrane AQP2 expression in rats with diabetes insipidus [22]. These findings suggest that aldosterone may decrease vasopressin-stimulated osmotic water permeability.

In this study, we investigated whether aldosterone affects vasopressin-stimulated osmotic water permeability in female and male rat IMCDs. We also investigated whether the aldosterone-mediated regulation of osmotic water permeability and urea permeability is through a genomic mechanism.

## 2. Materials and Methods

### 2.1. Animals

All animal surgical protocols and procedures were approved by the Emory Institutional Animal Care and Use Committee (protocol number "PROTO 201800110", approved on 21 December 2017, expires on 21 December 2020) and adhered to National Institutes of Health standards for animal use. These studies used both male and female rats from Charles River Laboratories, Wilmington, MA, USA. To measure osmotic water permeability (Pf) and urea permeability, rats weighing 50–75 g were sacrificed by decapitation to avoid any anesthesia effect, and the kidneys were quickly dissected to remove the inner medullas (IM). The rats used in this study were 3–4 weeks old. Urine concentrating ability is fully developed at that age, and the tubules are at good condition for dissection. To test AQP2 phosphorylation, rats weighing 100–140 g were sacrificed, and the IMs were placed on ice until tissue treatments.

### 2.2. Tubule Perfusion

IMs were transferred to a dissection dish, and the terminal IMCDs were microdissected in a dissection buffer at 17 °C. The dissecting solution contained (in mM): 125 NaCl, 25 NaHCO_3_, 2 CaCl_2_, 2.5 K_2_HPO_4_, 1.2 MgSO_4_, and 5.5 glucose. The solution osmolality was adjusted to 430 mOsmol/kg H_2_O with NaCl only for the purpose of dissecting tubules [23]. The perfusion and the bath solutions were identical to the dissection medium, except that 5 mM raffinose was added to both the perfusate and the bath, and an additional 70 mM NaCl was added to the bath when measuring osmotic water permeability to create a bath-to-lumen osmolality gradient of ~140 mOsmol/kg H_2_O. When measuring urea permeability, 5 mM raffinose was added to the perfusate, and 5 mM urea was added to the bath. All solutions were gassed continuously with 95% air and 5% CO_2_ before and during the dissection and the perfusion.

Single IMCDs were dissected, mounted on glass pipettes, and perfused as described [14]. In general, 45 min after warming the tubules to 37 °C in 2 mL of bath solution, two initial collections of 2–3 min at 6–8 nl/min were made. Treatments were added to the bath, and the tubules were allowed to equilibrate for 30 min; then, two further 2–3 min collections were made. To measure Pf, collected solutions were assayed for raffinose content by ultramicrofluorometry [24]; raffinose was used as a volume marker. To measure urea permeability, collected solutions were assayed for urea content by ultramicrofluorometry [14,25]. Pf and urea permeability were calculated as described [14]. Biological variation in baseline water permeability between different animals has been recognized for many years [26]. Therefore, we used each tubule as its own control.

To assess the contribution of aldosterone to vasopressin-stimulated osmotic water permeability, Pf was measured in the presence of vasopressin (50 pM), then aldosterone (500 nM) was added to the bath for 30 min, after which two perfusate collections were made for raffinose determination.

To determine whether the effect of aldosterone on vasopressin-stimulated osmotic water or urea permeability is a genomic action, actinomycin D (1 µM) was added to the bath to inhibit transcription after vasopressin-stimulated osmotic water or urea permeability were measured. Thirty minutes after addition of actinomycin D, aldosterone (500 nM) was added to the bath for a further 30 min, and osmotic water or urea permeability were measured.

### 2.3. Tissue Incubation

To measure AQP2 phosphorylation, one of the two IMs from the same animal was treated with vasopressin as a control; the other IM was treated with vasopressin and aldosterone. IMs were sectioned into ~1 mm^3^ tissue pieces. The IM pieces were first incubated at 37 °C for 15 min in isotonic Hanks’ balanced salt solution (HBSS) to establish base-line resting conditions, and then both the control and the treatment groups were stimulated for 20 min with 50 pM vasopressin. Subsequently, aldosterone (500 nM) was added to the treatment group, and incubation of all samples continued for 30 min. The reactions were terminated by placing the samples on ice and replacing the incubation solutions with ice cold homogenization buffer (10 mM triethanolamine, 250 mM sucrose, 10% sodium dodecyl sulfate). Tissues were homogenized and analyzed for AQP2 phosphorylation with western blot.

### 2.4. Western Blot Analysis

Samples of whole IM protein lysate (20 µg/lane), one rat per lane, were size-separated by SDS-PAGE on 12.5% gels and then electroblotted to polyvinylidene difluoride (PVDF) membranes (Immobilon, Millipore, Bedford, MA, USA). Blots were blocked with 5% nonfat dry milk in Tris-buffered saline (TBS: 20 mM Tris-HCl, 0.5 M NaCl, pH 7.5) and then incubated with primary antibodies overnight at 4 °C. Attached primary antibodies were identified using Alexa Fluor 680-linked anti-rabbit IgG (Molecular Probes, Eugene, OR, USA) and visualized using infrared detection with the LICOR Odyssey protein analysis system (Lincoln, NE, USA). Antibodies to AQP2 phosphorylated at serine 256 or 261 were purchased from Cell Signaling Technology (Danvers, MA, USA). Antibodies to AQP2 were made in our laboratory [24]. Beta tubulin was used to detect the loading levels of samples and was purchased from Abcepta (San Diego, CA, USA).

### 2.5. Statistics

All data are presented as means ± SE. Data from tubule perfusion studies were analyzed using a paired Student’s-t test [27]. Each perfused tubule serves as its own control, allowing a paired analysis of the data. Data from protein analysis studies were also analyzed using a paired Student’s t-test. The criterion for statistical significance is *P* < 0.05.

## 3. Results

### 3.1. Aldosterone Decreases Osmotic Water Permeability

**Figure 1:**
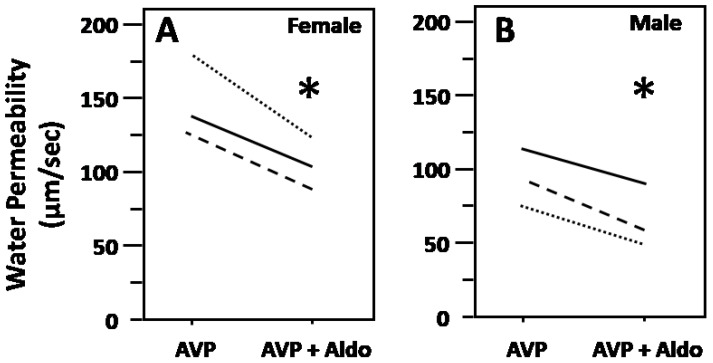
Aldosterone decreased vasopressin-stimulated osmotic water permeability in terminal inner medullary collecting ducts (IMCDs) from female (**A**) and male (**B**) rats. Terminal IMCDs were perfused with 50 pM vasopressin (AVP) for 20 min, samples were taken for analysis, then 500 nM aldosterone (Aldo) was added to the bath for 30 min, after which samples were taken for analysis. Each line represents a separate IMCD from a different rat. * *P* < 0.05 AVP control vs. AVP with Aldo, *N* = 3 rats/condition.

To determine whether aldosterone decreases Pf, male or female rat terminal IMCDs were first perfused with vasopressin and then perfused with aldosterone in the presence of vasopressin. Aldosterone decreased vasopressin-stimulated Pf from 146 ± 16 to 105 ± 10 µm/s in female rat IMCDs (*N* = 3, *P* < 0.05; Figure 1A) and from 92 ± 14 to 73 ± 10 µm/s in male rat IMCDs (*N* = 3, *P* < 0.05; Figure 1B). There was a statistically significant difference between the responses of male or female rats in osmotic water permeability (female average change: 41 ± 7 µm/s; male average change: 19 ± 3 µm/s, *P* < 0.05). To ensure that we were not observing a run-down phenomenon, we tested for time-related decreases in Pf in the presence of vasopressin and saw that vasopressin-stimulated Pf remained stable for up to 3 h (data not shown).

### 3.2. Aldosterone Phosphorylates AQP2

**Figure 2:**
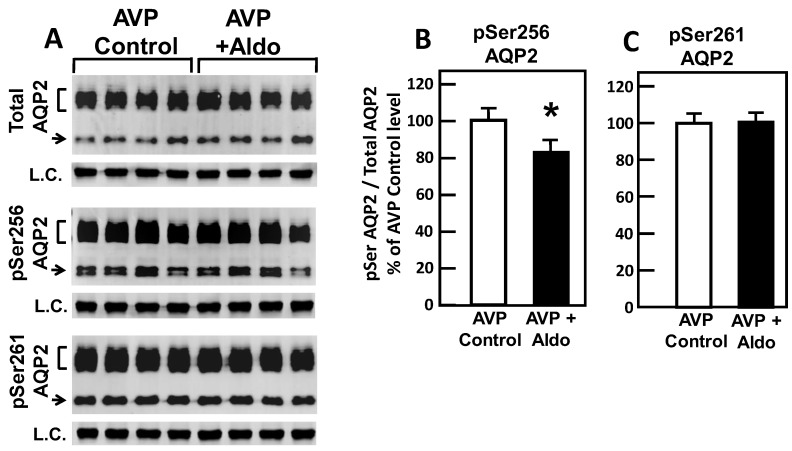
Aldosterone decreased phosphorylation of aquaporin-2 (AQP2) at serine 256 but did not change phosphorylation at serine 261 in male rat inner medulla (IM). (**A**) Representative western blots showing total AQP2 and phosphorylated AQP2 abundances in rat IM with AVP control vs. AVP plus Aldo for 30 min. Brackets indicate the glycosylated AQP2 protein between 35 and 45 kDa and arrow indicates the un-glycosylated AQP2 protein at 29 kDa. Beneath each AQP2 blot is the tubulin loading control blot (L.C.). Samples are from four different rats. The two kidney inner medullas from the same animal were randomly assigned into the AVP control or the AVP-Aldo groups. The matched pairs comparisons were achieved by comparing Lane 1 with Lane 5, Lane 2 with Lane 6, Lane 3 with Lane 7, and Lane 4 with Lane 8. (**B**) and (**C**) bar graph showing the ratio of pSer256 (**B**) or pSer261 (**C**) phosphorylated AQP2 to total AQP2 abundance as the percentage of the AVP-only control. Bars = means ± SE; * *P* < 0.05 AVP control vs. AVP with Aldo; *N* = 4 rats/condition.

To test the role of aldosterone in the phosphorylation of AQP2, IM tissues were incubated in isotonic medium for 20 min with vasopressin and then treated with aldosterone for 30 min. Tissue lysates were analyzed by western blot, probing for total AQP2, pSer256 AQP2, and pSer261 AQP2 (Figure 2A). Aldosterone significantly decreased the amount of AQP2 that was phosphorylated at serine 256 by 17% (AVP control: 1.35 ± 0.05, aldosterone-treated: 1.11 ± 0.07, *N* = 4, *P* < 0.05; Figure 2B). However, aldosterone did not significantly change the phosphorylation of AQP2 at serine 261 (AVP control: 1.30 ± 0.04, aldosterone-treated: 1.34 ± 0.04, *N* = 4, *P* < 0.05; Figure 2C).

**Figure 3:**
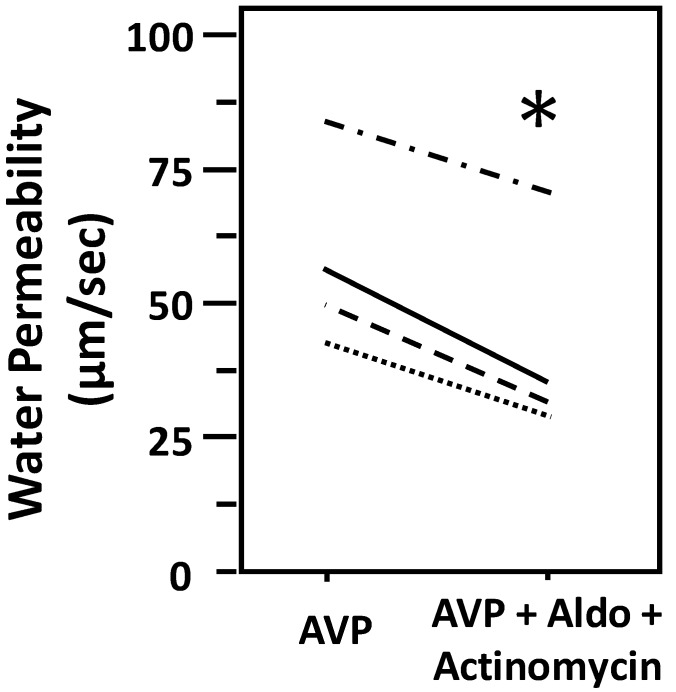
Aldosterone-attenuated osmotic water permeability in male rat terminal IMCDs was not reversed by inhibition of gene transcription with actinomycin. Terminal IMCDs were perfused with 50 pM vasopressin (AVP), then treated with 1 µM actinomycin for 30 min before adding 500 nM aldosterone (Aldo) for 30 min. Each line represents a separate IMCD from a different rat. * *P* < 0.05 AVP control vs. AVP with Aldo plus actinomycin, *N* = 4 rats/condition.

Aldosterone decreases osmotic water permeability in the presence of actinomycin D. To test whether aldosterone-attenuated osmotic water permeability through a genomic mechanism, IMCDs were first perfused with vasopressin, then perfused with actinomycin D in the presence of vasopressin, and finally perfused with aldosterone in the presence of vasopressin and actinomycin D. Aldosterone decreased vasopressin-stimulated Pf from 55 ± 10 to 42 ± 9 µm/s (*N* = 4, *P* < 0.05; Figure 3). There was no significant difference in aldosterone-inhibited osmotic water permeability regardless of the presence of actinomycin D (with actinomycin D: 15 ± 3 µm/s, without actinomycin D: 19± 3 µm/s, *P* > 0.05).

**Figure 4:**
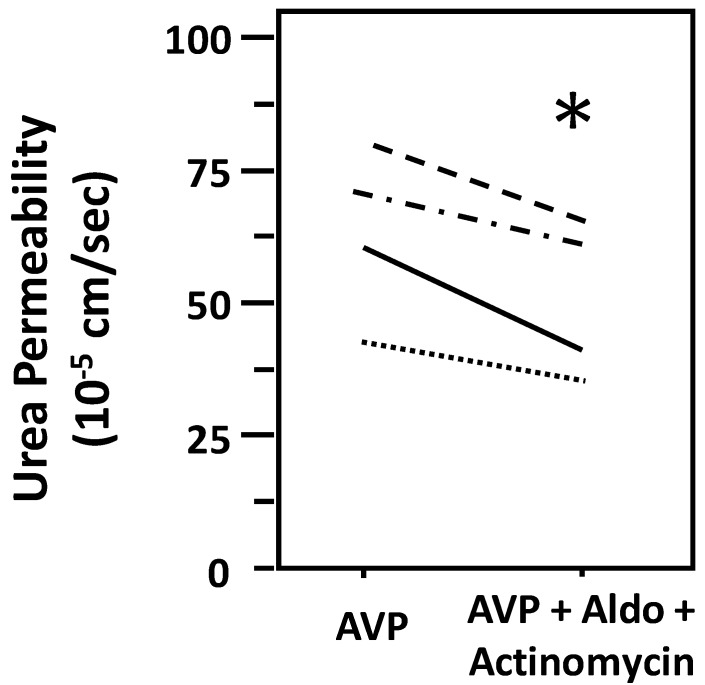
Aldosterone-attenuated urea permeability in male rat terminal IMCDs was not reversed by inhibition of gene transcription with actinomycin. Terminal IMCDs were perfused with 50 pM vasopressin (AVP), then treated with 1 µM actinomycin for 30 min before adding 500 nM aldosterone (Aldo) for 30 min. Each line represents a separate IMCD from a different rat. * *P* < 0.05 AVP control vs. AVP with Aldo plus actinomycin, *N* = 4 rats/condition.

Aldosterone decreases urea permeability in the presence of actinomycin D. To test whether aldosterone-attenuated urea permeability through a genomic mechanism, IMCDs were first perfused with vasopressin, then perfused with actinomycin D in the presence of vasopressin, and finally perfused with aldosterone in the presence of vasopressin and actinomycin D. Aldosterone decreased vasopressin-stimulated urea permeability from 63 ± 8 to 51 ± 7 × 10^-5^ cm/s (*N* = 4, *P* < 0.05; Figure 4). We tested for a time-related decrease in urea permeability in the presence of vasopressin and saw that vasopressin-stimulated urea permeability remained stable for up to 3 h (data not shown).

## 4. Discussion

Water transport is regulated by vasopressin through cAMP-dependent signaling pathways. Along with vasopressin, aldosterone is involved in the regulation of body fluid homeostasis. Aldosterone increases sodium transport by binding to the MR, translocating to the nucleus, and increasing the transcription of sodium channels and transporters, thereby indirectly promoting water reabsorption in the renal distal tubule. However, recent studies demonstrate that aldosterone may play a different role in water transport in other renal tubules [21,22]. This study provides evidence that aldosterone directly and rapidly decreases vasopressin-stimulated osmotic water and urea permeability. Furthermore, the effect of aldosterone on vasopressin-stimulated osmotic water and urea permeability is a non-genomic action. In this study, we used a different model from those employed by the Nielsen group that investigated the effects of aldosterone in polyuric conditions, where AVP was either blocked (lithium-NDI) or AVP was not present (Brattleboro rat) [28]. Our data show that aldosterone has an inhibitory effect on water reabsorption in rat IMCD, thus we anticipate that, in a polyuric animal model, aldosterone would exacerbate the water imbalance of the animals.

Previous studies show aldosterone increases urine production and decreases apical AQP2 expression in rats with diabetes insipidus, suggesting that aldosterone may decrease vasopressin-stimulated osmotic water permeability [13,22]. There are some sex-specific differences in blood pressure control and water homeostasis (reviewed in [29]). However, whether there is a sex difference in the response to aldosterone in perfused rat IMCDs has never been studied. Our data show that aldosterone significantly decreases osmotic water permeability in both female rat IMCDs (Figure 1A) and male rat IMCDs (Figure 1B). Furthermore, it appears that there may be a difference in response to aldosterone inhibition between the female and the male rats. However, further experiments need to be performed to examine whether the female rat is more susceptible to the inhibition of osmotic water permeability by aldosterone than is the male rat.

Phosphorylation of AQP2 at serine 256 promotes AQP2 trafficking to the apical plasma membrane [30], and phosphorylation of AQP2 at serine 261 is linked to ubiquitination [31], which is consistent with the finding that aldosterone increases urine production and decreases apical AQP2 expression in rats with diabetes insipidus [22]. Our data indicate that aldosterone decreases AQP2 phosphorylation at serine 256 (Figure 2B) in the presence of vasopressin, which may decrease AQP2 accumulation in the apical membrane. There was no observed change in the phosphorylation of serine 261 AQP2 (Figure 2C). Thus, the decrease in vasopressin-stimulated water permeability by aldosterone may be related to the decrease in AQP2 trafficking to the plasma membrane rather than an increase in AQP2 endocytosis. However, Kwon et al. indicated that short-term aldosterone treatment does not alter the intracellular distribution of AQP2 in rats with diabetes insipidus [32]. The inconsistency in the findings may be due to the different animal models used. Contrary to its stimulatory role in water reabsorption in the distal tubule, our results indicate that aldosterone has a direct and rapid inhibitory effect on water reabsorption in IMCDs. The discrepancy in the role of aldosterone suggests that there may be different pathways stimulated by aldosterone in the different cell types in distal tubules versus IMCDs. We will test whether there are sex differences in the trafficking of AQP2 and endocytosis of AQP2 after aldosterone treatment in the future.

Aldosterone exerts effects through both genomic and non-genomic actions. In distal tubules, aldosterone regulates sodium and water reabsorption through a genomic mechanism, i.e., translocation of the MR complex to the nucleus [2]. This alters the transcription of key transporters and transport regulators, such as kinases, and results in changes in the activity of several membrane transporters. In the case of aldosterone, the most notable of these transporters is ENaC, which is activated to increase sodium reabsorption in the cortical collecting duct [33]. Our data using actinomycin D, an inhibitor of gene transcription, suggest that aldosterone has a different, non-genomic effect in the IMCD. Aldosterone significantly decreased vasopressin-stimulated osmotic water permeability in the presence of actinomycin. This was not significantly different from that in the absence of actinomycin, suggesting that all changes in osmotic water permeability in response to aldosterone ascribe to a non-genomic pathway. In addition, our data show that aldosterone inhibited water reabsorption in 30 min. Since activation of genomic mechanisms is highly unlikely to happen in such a short period, we surmise that aldosterone rapidly inhibits water absorption initially by a non-genomic pathway. We did not explore the inhibitory effect of aldosterone on vasopressin-stimulated water permeability beyond 2 h due to the limitation of the isolated tubule perfusion technique. Interestingly, Hasler et al. showed that aldosterone reduced AQP2 mRNA and protein expression in mpkCCDc14 cells when applied together with vasopressin for a short period (≤24 h). Nine-hour treatment with actinomycin D prevented aldosterone-induced downregulation of AQP2 protein in vasopressin-treated cells. Hasler’s findings indicate that a genomic mechanism is subsequently activated to strengthen the inhibitory effect of aldosterone in 24 h. Hasler et al. also showed that, for a longer period (48 h), aldosterone increased AQP2 expression despite sustained low levels of AQP2 mRNA [34]. A longer incubation with aldosterone might activate a variety of mechanisms to modify homeostasis in the IMCD, but this is beyond the scope of our study.

Urea is also crucial for the urinary concentrating mechanism (reviewed in [35]). Vasopressin stimulates both osmotic water and urea permeability through the cAMP-PKA pathway to maintain urine concentrating ability [14,20,36]. To examine whether aldosterone also decreases urea permeability and whether the inhibition by aldosterone is through a genomic action, IMCDs were perfused with vasopressin in the presence of actinomycin D. Our data show that aldosterone significantly decreases vasopressin-stimulated urea permeability in the presence of actinomycin D, suggesting that the aldosterone-attenuated urea permeability is a non-genomic action. However, we cannot exclude the possibility that effects are attributed to aldosterone and not to actinomycin D based on the current data. The consistency of the results for aldosterone-regulated water and urea permeability suggest that aldosterone can decrease urine concentrating ability in a rapid manner that does not involve genomic changes.

In conclusion, the present study shows that aldosterone is an independent regulator of water permeability. Aldosterone decreases vasopressin-stimulated water transport in both male and female rats. The decrease in water permeability appears to be a result of a decrease in AQP2 phosphorylation at serine 256, which may decrease AQP2 accumulation at the plasma membrane. Aldosterone-attenuated osmotic water and urea permeability are non-genomic processes. This is the first report of aldosterone directly affecting vasopressin-stimulated water and urea transport in the IMCD.
